# Evaluating the Asymmetry of Muscle Activation and Strength in Paralympic Powerlifting Athletes

**DOI:** 10.3390/ejihpe13090119

**Published:** 2023-09-01

**Authors:** Leonardo dos Santos, Felipe J. Aidar, Rafael Luiz Mesquita Souza, Dihogo Gama de Matos, Stefania Cataldi, Gianpiero Greco, Márcio Getirana-Mota, Anderson Carlos Marçal, Lucio Marques Vieira Souza, Jymmys Lopes dos Santos, Paulo Francisco de Almeida-Neto, Breno Guilherme de Araújo Tinoco Cabral, Georgian Badicu, Hadi Nobari, Raphael Frabrício de Souza

**Affiliations:** 1 Graduate Program of Physical Education, Federal University of Sergipe (UFS), São Cristovão 49100-000, Brazil; leoprofedf@gmail.com (L.d.S.); marcio_getirana@hotmail.com (M.G.-M.); acmarcal.ufs@gmail.com (A.C.M.); profedf.luciomarkes@gmail.com (L.M.V.S.); jymmys.lopes@gmail.com (J.L.d.S.); raphaelctba20@hotmail.com (R.F.d.S.); 2 Group of Studies and Research of Performance, Sport, Health and Paralympic Sports (GEPEPS), Federal University of Sergipe (UFS), São Cristovão 49100-000, Brazil; rlms2010@hotmail.com; 3 Department of Physical Education, Federal University of Sergipe (UFS), São Cristovão 49100-000, Brazil; 4 Graduate Program of Physiological Science, Federal University of Sergipe (UFS), São Cristovão 49100-000, Brazil; 5 Cardiovascular & Physiology of Exercise Laboratory, University of Manitoba, Winnipeg, MB R3T 2N2, Canada; dihogogmc@gmail.com; 6 Department of Translational Biomedicine and Neuroscience (DiBraiN), University of Study of Bari, 70124 Bari, Italy; stefania.cataldi@uniba.it (S.C.); gianpiero.greco@uniba.it (G.G.); 7 Department of Physical Education, Federal University of Rio Grande do Norte (UFRN), Natal 59078-970, Brazil; paulo220911@hotmail.com (P.F.d.A.-N.); brenotcabral@gmail.com (B.G.d.A.T.C.); 8 Department of Physical Education and Special Motricity, Transilvania University of Brasov, 500068 Brasov, Romania; georgian.badicu@unitbv.ro; 9 Faculty of Sport Sciences, University of Extremadura, 10003 Cáceres, Spain; hadi.nobari1@gmail.com

**Keywords:** disabled persons, asymmetry, force, paralympic sports

## Abstract

Background: Strength training is a complex task, as it requires a combination of many variables. In paralympic powerlifting (PP) asymmetries for the evaluation of activation, and static force indicators have been increasingly studied. Objective: To investigate the asymmetries in the strength and muscle activation indicators, before and after a training session of PP athletes. Methodology: Twelve elite athletes from the PP participated in the study, and asymmetry was evaluated through surface electromyography (sEMG) and static strength indicators. Evaluations were made before and after a protocol of five series of five repetitions (5 × 5), with 80% of 1-Maximum Repetition (1RM). Results: In the pectoral muscles, there were differences in the non-dominant limbs between the before and after in the sEMG. There were differences in the pectoralis muscle in the non-dominant limb between moments before (110.75 ± 59.52%) and after (130.53 ± 98.48%, *p* < 0.001), and there was no difference in triceps activation. In the Maximum Isometric Strength (MIF), there was a difference in the non-dominant limb between before (710.36 ± 129.36) and after (620.27 ± 69.73; *p* < 0.030). There was a difference before in the dominant (626.89 ± 120.16; 95% CI 550.55–703.24) and non-dominant (710.36 ± 129.36; *p* = 0.011) limbs. There was no difference in time to MIF. Conclusion: PP athletes showed small levels of asymmetry before and after training, and adaptation to training tends to promote fewer asymmetries.

## 1. Introduction

Strength training is a complex task, as it requires a combination of many variables (such as exercise selection, execution order, number of repetitions, sets, and, loads) [1]. Evaluating and controlling training are directly related to performance and injury prevention. The observation of muscular activity through electromyography has established a reliable relationship with evaluating acute training responses [2]. In strength training, one of the most utilized exercises for developing upper limb strength is the Bench Press, which is also a piece of equipment in modalities such as Conventional and Paralympic Powerlifting [3,4,5]. In Paralympic Powerlifting (PP), the bench press is the only competition exercise [6].

The presence of asymmetries between the limbs has been studied, with few studies specifically addressing performance [7]. Similarly, it has been reported that limb dominance influences movement kinematics, or is influenced by specific activity, as certain modalities place greater demands on one side of the body and highlight the dominant side compared to the non-dominant limb [8]. Thus, the dominant limb exerts greater control in more stable mechanical actions, while the non-dominant limb provides greater stability in unpredictable actions. Maximum voluntary force showed a difference between the dominant and non-dominant sides, with the average being higher on the non-dominant side [9].

The increase in asymmetries would be related to the increased risk of injuries [10,11]. Thus, training load control has been important in reducing the risk of injuries and improving performance [1,12]. On the other hand, fatigue would be related to loss of speed and metabolic stress [12,13]. Allied with this, the decrease in asymmetries would be related to improvements in function, strength, and power. Thus, studies have indicated that different neural control mechanisms are employed for dominant and non-dominant arm movements [14]. The neuromuscular control would be the muscular response to a stimulus linked to the dynamic stability of one or more joints. These neurological messages relate to different aspects of muscle actions, coordination, stabilization, posture, and balance [15]. Thus, fatigue could compromise these interactions and interfere with balance and symmetry. On the other hand, training would have effects on functional performance, biomechanics, and muscle activation patterns of the surrounding joint musculature. [14,15,16], and this asymmetry could be related to an increased risk of injuries [17,18].

One way to assess Bench Press and muscle activation would be through electromyography, where the activation of the muscles involved in the exercise tends to be better researched [19,20,21]. Evaluations have been used to identify the recruitment of muscles involved in exercises, muscle activation, the effect of different training methods, and asymmetry, among others [3,22,23]. In addition to the evaluation in terms of activation, several studies have focused on the assessment through dynamic and static force indicators [24,25]. In addition, the assessment of asymmetries in relation to muscle activation [26,27,28] and static strength indicators [9,29] has been widely used.

Thus, the aim of this study was to compare the asymmetry before and after a training session in PP athletes in relation to muscle activation and static strength indicators. For this, it was hypothesized that there will be differences in relation to muscle activation and static strength indicators in relation to the dominant and non-dominant side between PP athletes.

## 2. Materials and Methods

### 2.1. Design

**Figure 1:**
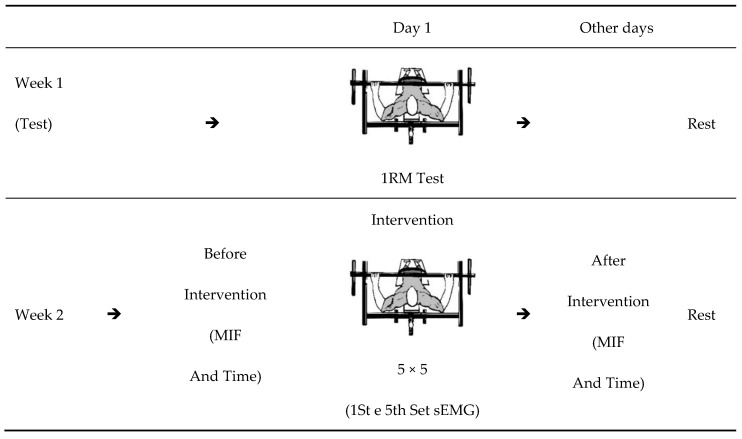
Experimental design. Legend: MIF: Maximum Isometric Force. Time: Time at MIF. The sEMG: surface electromyography. 5 × 5: five sets of five maximum repetitions.

The study was carried out over two weeks, with the first week focused on familiarizing and testing a maximum repetition (1RM) in the Bench Press (BP) exercise. Before and after the training session, the athletes were submitted to a test to evaluate the Maximum Isometric Strength (MIF) and time in milliseconds for MIF (time). The training session was performed using a protocol of five series of five repetitions (5 × 5) with 80% 1RM in the BP exercise. To assess muscle activity, it was performed using surface electromyography (sEMG), and surface electrodes were applied in the first and last series of the 5 × 5 protocol [23,24], to collect the electromyographic activity of the Pectoralis Major (PM) muscles, (sternal portion) and Triceps Brachii (TB) long head (Figure 1).

The tests were carried out on Monday, from 8:00 a.m. to 12:00 p.m., and the athletes were instructed to maintain their eating habits, not to consume alcohol, and not to practice physical activities in the 48 h that preceded the tests. This was confirmed by interviewing the athletes before the tests.

### 2.2. Sample

Twelve elite male athletes from PP (age: 27.7 ± 5.7 years; experience: 2.1 ± 0.9 years; body mass: 74.0 ± 19.5 kg; 1RM: 113.0 ± 31.3 kg; 1RM/Body Mass: 1.6 ± 0.3), all athletes were right-handed, linked to an extension project of the Physical Education Department of the Federal University of Sergipe—Sergipe—Brazil. All participants have competed at the national level, are eligible for the modality competition [6], and share a ranking among the top ten in their respective categories. Among the disabilities: six athletes have malformation in the lower limbs (arthrogryposis); one with sequelae due to poliomyelitis; four with amputations; and one with a spinal cord injury due to an accident with injury below the eighth thoracic vertebra. The athletes participated in the study voluntarily and signed an informed consent form, according to resolution 466/2012 of the National Commission of Ethics in Research—CONEP, of the National Health Council, in accordance with the ethical principles expressed in the Declaration of Helsinki (1964, reformulated in 1975, 1983, 1989, 1996, 2000, 2008, and 2013), of the World Medical Association. The project was submitted and approved by the Research Ethics Committee of the Federal University of Sergipe with the number Certificate of Presentation of Ethical Appreciation (CAAE): 2,637,882 (approval date: 7 May 2018).

### 2.3. Instruments/Procedures

Individual body mass of the athletes was obtained using a digital electronic platform scale (Michetti, São Paulo, Brazil) with a maximum weight capacity of 300 kg and dimensions of 1.50 × 1.50 m to facilitate weighing while seated. For the execution of the bench press exercise, an official flat bench measuring 210 cm, a 220 cm barbell, and weight plates from the brand Eleiko (Eleiko, Halmstad, Sweden) were used, all approved by the International Paralympic Committee [6].

Prior to the 1RM test in the first session and the 5 × 5 protocol with 80% of 1RM in the second session, the athletes performed a specific warm-up for upper limbs on the bench press itself with 30% of 1RM, where 10 slow repetitions (3.0 × 1.0 s, eccentric × concentric) and 10 fast repetitions (1.0 × 1.0 s, eccentric × concentric) were performed, followed by data collection [30]. In the first session, the 1RM test was performed, where each subject started the attempt with a weight, they believed they could lift only once using maximum effort. Weight increments were added until the maximum load that could be lifted was reached. If the athlete was unable to perform a single repetition, 2.4 to 2.5% of the weight used in the test was subtracted [31,32,33,34]. The subjects rested between 3–5 min between attempts [22,32]. This test was conducted 72 h prior to the evaluative process that occurred in the second session.

**Figure 2:**
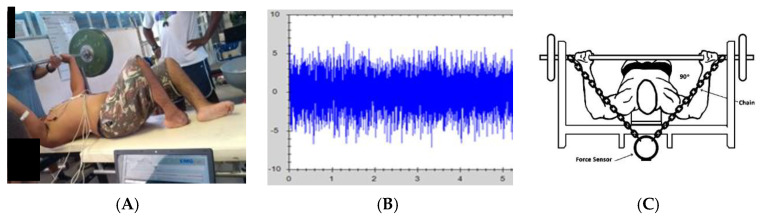
(**A**) Demonstration of muscle activation assessment using surface electromyography (sEMG); (**B**) sEMG signal; and (**C**) Demonstration of force sensor fixation.

On the second day, the tests of MIF and time to MIF between the right and left sides of the upper limbs were performed before and after the training. The data for these indicators were determined by a Force Sensor (Chronojump, Boscosystem, Barcelona, Spain), with a capacity of 500 kg and an output impedance of 350 ± 3 ohm, fixed on the adapted Flat Bench Press (Figure 2), through the use of Spider HMS Simond model carabiners (Simmond, Chamonix, France), with a breaking load of 21 KN, approved for climbing by the Union Internationale des Associations d’Alpinisme (UIAA). A steel chain with a breaking load of 2300 kg was used to fix the load cell to the bench. The perpendicular distance between the load cell and the joint center was determined and used to calculate the results of the indicators on both sides of the body [35,36,37].

The electromyographic activity of the muscles was captured using a New Mio Miotool (Miotec Inc. Porto Alegre, Brazil), and the surface electrodes were double, bipolar, and disposable type. They were applied on the right and left sides of the PM (sternal portion) and TB (long head), and the grounding electrode was fixed on the olecranon, according to surface EMG for a non-invasive assessment of muscles (SENIAM) recommendations [38]. Before the tests and electrode application, local scraping and cleaning were performed, and then the measurement points of each muscle were marked with a felt-tip pen. High-pass and low-pass filters (500-20) and offset were used for signal rectification. The highest signal and root mean square (RMS) were used for signal presentation, and signal normalization was performed based on the maximum voluntary isometric contraction (MVIC) [22,32] (Figure 2).

The maximum voluntary isometric contraction (MVIC) of each muscle was then recorded to normalize the sEMG values that would be recorded in the bench press exercise. For this, two maximal isometric contractions were performed for 3 s with 10 s of rest between contractions [39] and 2 min between MVIC assessment of each muscle [40]. Specifically, the MVIC was performed as follows: For the PM and TB, the participants performed bench presses with a grip that would do the training. sEMG targeted the sternal portion of the pectoralis major (PSL) and the long head of the triceps brachii (LHTB), which were captured during 3-s maximal voluntary isometric contractions (MVIC). There was an elbow angulation of approximately 90°, with a distance of 15.0 cm from the bar to the stern [23]. The surface electrodes (double, bipolar, and disposable) were positioned at an average distance between the motor point and the tendon of the evaluated muscles, parallel to the muscle fibers with a distance of 20 mm between them. A reference electrode fixed to the olecranon was also used, as recommended by SENIAM (Surface Electro Myo Graphy for the non-invasive assessment of muscles) [38]. Before the test, local asepsis and trichotomy were performed, and then the measurement points were marked with a felt-tip pen.

The entire adapted bench press test was accompanied by an experienced professional who instructed the athletes to perform the efforts symmetrically and quickly impose a stop at the chest between the eccentric and concentric phases, and equal locking of the elbows [6].

### 2.4. Statistics

Descriptive statistics were performed using measures of central tendency, mean (X) ± standard deviation (SD), and 95% confidence interval (95% CI). The Shapiro-Wilk test was used to check for the normality of the variables given the sample size. Data from all analyzed variables were found to be homogenous and normally distributed. Two-way repeated-measures ANOVA and Bonferroni post-hoc tests were conducted to evaluate the differences between pre- and post-training as well as between the right and left sides. The level of significance was set at *p* < 0.05. Effect size (partial eta-squared: η2p) was used to determine the magnitude of the differences, with values of low effect (≤0.05), medium effect (0.05 to 0.25), high effect (0.25 to 0.50), and very high effect (>0.50) [41,42]. All statistical analyses were performed using the Statistical Package for the Social Science (SPSS) software, version 22.0 (IBM, New York, NY, USA).

## 3. Results

**Figure 3:**
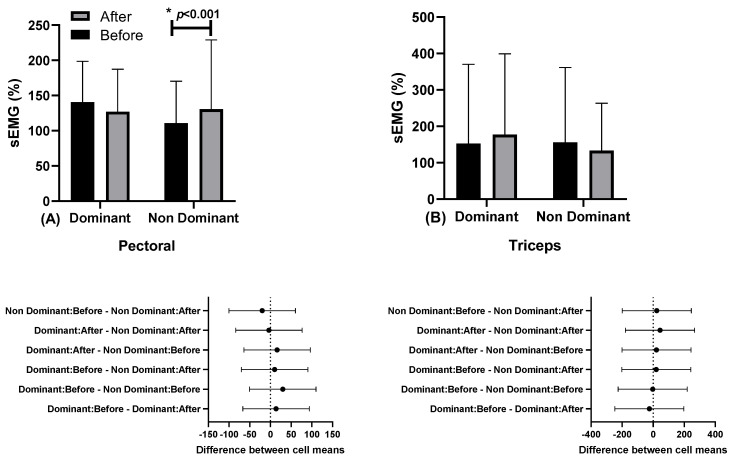
Muscle activation through surface electromyography (sEMG) in the pectoralis, and below in the same column is the 95% confidence interval (**A**) and triceps (**B**) muscles, in dominant and non-dominant limbs, at moments before (series 1) and after (series 5), with a load of 80% 1RM.

In Figure 3, the results of muscle activation through surface electromyography (sEMG) in the pectoral and triceps muscles are presented.

There were differences in the pectoralis muscle in the non-dominant limb between moments before (110.75 ± 59.52%; 95% CI 72.82–148.57) and after (130.53 ± 98.48%, 95% CI 67.96–193.11; “*” *p* < 0.001; η2p = 0.131, medium effect). No differences were observed for the triceps brachii muscle.

**Figure 4:**
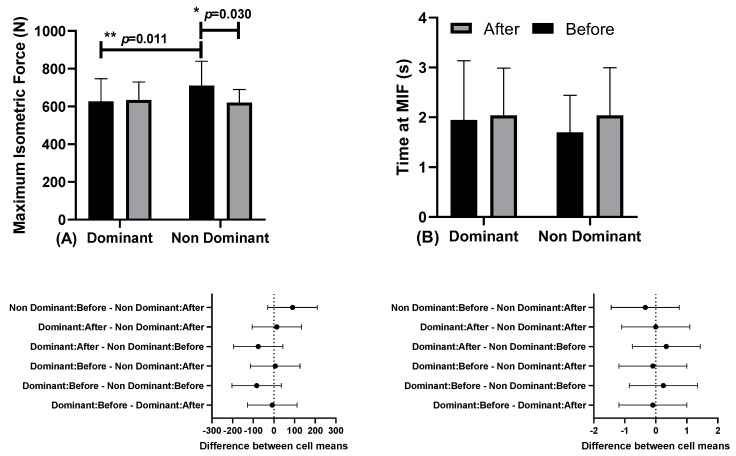
(**A**) Maximum Isometric Force (MIF) and (**B**) time to MIF, and below in the same column is the 95% confidence interval, before and after moments, in relation to dominant and non-dominant limbs.

Figure 4 shows the results of maximum isometric force (MIF) and time to MIF, at moments before and after, in relation to the dominant and non-dominant limbs.

There were differences in Maximum Isometric Force (MIF) in the non-dominant limb between the before moment (710.36 ± 129.36 N; CI 95% 628.17–792.55) and after moments (620.27 ± 69.73 N; CI 95% 575.96–664.57; “*” *p* < 0.031; η2p = 0.357, medium effect). There were also differences at the before moment between dominant (626.89 ± 120.16; CI 95% 550.55–703.24) and non-dominant limbs (710.36 ± 129.3 N; CI 95% 628.17–792.55; *p* = 0.011; η2p = 0.376, high effect). No differences were observed for the Time at MIF.

## 4. Discussion

The aim of this study was to investigate muscular activity asymmetries in different sets of the bench press exercises performed by powerlifters. We hypothesized that there were differences between members across different grades. In the analysis of sEMG in the non-dominant limb, an increase in activation was observed, however, there were no asymmetries in the PM and TB muscles.

The evidence from our study, which showed differences in the normalized average activity of electromyography (sEMG) in the PM muscle favoring the non-dominant limb in the post-exercise moment, may be related to greater sEMG signals from this area compared to other muscle segments, given that in the SP movement, the sternal and clavicular portions of the pectoral muscle are more solicited [43].

Understanding the last set as a more unstable means due to fatigue, this finding can also be explained by the Dynamic Dominance Model, in which in unexpected actions, such as fatigue, the non-dominant limb would be more activated than the dominant limb [8]. It was observed that during the assessment of maximal voluntary force when evaluating muscle actions, the non-dominant limb exhibited greater force than the contralateral limb [9]. Our study observed that activation in the Pectoralis Major muscle, on the non-dominant side afterwards, corroborates with other studies that presented similar results regarding the non-dominant side and recruitment patterns [9,44]. In addition to the aforementioned, the Pectoralis Major also showed greater asymmetry, especially with higher intensities (80% 1RM), where it was observed that the asymmetry would be specific to the mentioned muscle [8,14].

It is worth noting that the sEMG in the PM was higher in the post-exercise moment and on the non-dominant side, an effect explained by the logic of the recruitment progression of motor units (MU) [44]. The non-dominant PM exhibited greater muscle activity in the post-exercise moment, a behavior that suggests a different MU recruitment pattern between the limbs as the exercise series progressed in the SP at an intensity of 80% of 1RM.

When comparing the peak amplitude of sEMG in the PM, and TB during the bench press exercise at 50% and 90% of 1RM performed until muscular failure, investigators found different results from our study, showing significant sEMG differences only for the TB [45]. Another study aimed to analyze the inter-limb asymmetry index (IS) of sEMG in a group of powerlifting paraplegics, and they found significant IS for the pectoralis major, with a predominance for the right side [3].

Regarding the TB, they did not find statistical differences, but absolute values were highlighted due to an interesting particularity, as greater asymmetry was found on the left side, exceeding double that of the opposite side, a data that was not observed among other subjects in the study [3]. These findings indicate that muscle activity is complex and individual, dependent on load, repetition, and set quantity, making these variables important in evaluating differences in muscle activity between body sides.

Muscular strength is an important physical component that has been investigated by several studies [16,19,23] and among its indicators, the 1RM is considered the most reliable and efficient way to assess maximum strength [46]. As our sample was specialized in PP, a modality in which the development of maximum strength is a crucial element [6], we tested the 1RM and the time in milliseconds to reach it on each side of the limbs. To better understand muscle activity in the BP, our 1RM results showed significant differences in the non-dominant limb and at different moments. This reinforces the assumption that the dominant limb is more engaged in anticipating and dynamically controlling the movement, while the non-dominant limb performs the action of maintaining the position [15,16]. Regarding the time in milliseconds until the 1RM, there was no significant difference.

Another study evaluating grip widths of 1×, 1.3×, and 1.5× biacromial distance (BD) did not find significant differences in MIF [30]. The intersection of our findings with the results of this research enriches the interpretation of the interlimb training effect, as well as the effort generated after sets of the exercise. That is, the MIF of the upper limbs in SP does not change with different grip widths, but it is reflected differently in relation to the sides of the body.

The results regarding the time to reach MIF did not show significant differences between sides or between moments before and after. Under conditions of training with partial and total range of motion, this variable also did not undergo significant changes [23]. It is worth noting that this study did not evaluate the inter-limb effect of different training methods, but by combining our evidence, we contribute to understanding the behavior of these variables in the BP exercise.

Due to a multiplicity of reasons, para-athletes experience different levels of strength between the sides of the body (asymmetry) [47]. Time and specific activity make this asymmetry natural and individualized, and it is not determinant in compromising performance [48]. On the other hand, studies indicate that excessive asymmetries may be related to injury risks [10,11] and are also not accepted by the rules that govern PP competitions [6]. On the other hand, new studies are necessary to evaluate other perspectives, such as soft tissue radiodensity [49,50], functional electrical stimulation [51,52], the use of stimuli through stretching [53], and the combination of training [54].

Our study has certain limitations, including the lack of control over the athletes’ diet and sleep patterns. Additionally, the research sample consisted of a small group of national and international level athletes who may have adapted to training and displayed less asymmetry than the general population due to the rules of the sport that prohibit asymmetry during lifts. We did not evaluate the relationship between disabilities and potential asymmetries, as many athletes had injuries on only one side of their body.

## 5. Conclusions

Our findings suggest that Paralympic powerlifting athletes showed very small levels of asymmetry and, even after training, this asymmetry was not observed in muscle activation through sEMG. With regard to FIM, there was asymmetry in the moment before, but not after training. While there were differences in terms of the pectoralis major and FIM muscle activation between the dominant and non-dominant sides, there was no asymmetry in terms of triceps FIM activation. Therefore, it seems that the PP athletes showed good symmetry, and even after training with high intensities, asymmetries were not observed in these athletes.

## Data Availability

The data that support this study can be obtained from the address: www.ufs.br/DepartmentofPhysicalEducation, accessed on 12 June 2023.
